# Fabrication of transition metal dichalcogenides quantum dots based on femtosecond laser ablation

**DOI:** 10.1038/s41598-019-38929-5

**Published:** 2019-02-27

**Authors:** Yanmin Xu, Lihe Yan, Xiaoyu Li, Huanhuan Xu

**Affiliations:** Key Laboratory for Physical Electronics and Devices of the Ministry of Education and Shaanxi Key Lab of Information Photonic Technique, School of Electronics and Information Engineering, Xi’an Jiaotong University, Xi’an, 710049 China

## Abstract

As heavy metal-free quantum dots, transition metal dichalcogenides (TMDs) and boron nitride (BN) quantum dots (QDs) have aroused great interest due to features such as good thermal conductivity, chemical stability, and unique optical properties. Although TMDs have been synthesized using different methods, most of these methods require time-consuming or complex steps, limiting the applications of TMDs. We propose a fast and simple method for the synthesis of high-quality molybdenum disulfide (MoS_2_) QDs and tungsten disulfide (WS_2_) QDs based on femtosecond laser ablation and sonication-assisted liquid exfoliation. The prepared MoS_2_ QDs and WS_2_ QDs were characterized by transmission electron microscopy, atomic force microscopy, X-ray photoelectron spectroscopy, and Fourier transform infrared spectroscopy. The resulting products possessed few-layered thickness with an average size of 3.7 nm and 2.1 nm. Due to the abundance of functional groups on their surface, the MoS_2_ QDs and WS_2_ QDs showed bright blue-green luminescence under UV irradiation. Our method offers a facile and novel synthetic strategy for TMDs QDs and other two-dimensional nanomaterial quantum dots, such as boron nitride quantum dots (BNQDs).

## Introduction

Following the successful application of graphene, great attention has been paid to other layered inorganic graphene analogues due to their peculiar and fascinating physical properties that are correlated with their 2D ultrathin atomic layer structure. Transition metal dichalcogenides (TMDs) have attracted increasing attention in recent years due to their unique optical and electronic properties and have found many applications in catalysts, optoelectronics, and bio-imaging^1–4^. As the electronic band structure of semiconductor materials is relatively sensitive to the quantum size effect, layered TMDs exhibit excellent fluorescence properties when they are tailored into quantum dots (QDs)^5–8^. Compared with traditional semiconductor QDs (such as CdS and CdSe), TMDs QDs have been proven to be good candidates for bio-imaging and bio-sensing areas due to their intrinsic low toxicity and good dispersibility^2,9–11^.

Similar to the fabrication of well-known carbon QDs or carbon nanodots (C-dots), the synthetic strategies of TMDs can be divided into two groups: top-down and bottom-up methods. Top-down methods mainly use physical or chemical methods to weaken the van der Waals forces between the layers and tailor them into QDs. Although monolayered TMDs can be made by ultrasonication^12^, intercalation reaction^11,13^ and CVD methods^14^, further reduction of the lateral size of TMDs film to form QDs has remained a significant challenge. For example, P. Wu *et al*. fabricated MoS_2_ and WS_2_ QDs with controllable size using the sonication-assisted liquid exfoliation technique followed by a solvothermal process that was carried out at 140 °C for 9 h^7^. Although TMDs QDs have been successfully synthesized by top-down methods, the top-down preparation processes of TMDs QDs is generally time-consuming. In contrast with the top-down methods, the bottom-up methods involve the oxidative condensation of different elements, which is typically used to produce C-dots on the basis of dehydrogenation and carbonization^15–17^. Due to the difficulties in selecting proper precursors, much less attention has been devoted to TMDs QDs synthesized by the bottom-up methods. W. Song *et al*. obtained MoS_2_ QDs by hydrothermal treatment of a mixture of ammonium molybdate and thiourea. However, their further application was largely hindered because the ammonia solution was harmful to human tissue^18^. Based on the above reasons, it is necessary to develop a new fast, green and facile method for preparing TMDs QDs.

Femtosecond laser ablation has attracted much attention due to its outstanding features, such as being fast, clean and efficient^19,20^. When the femtosecond pulses are injected into the targets, multiphoton-absorption ionization occurs, and a plasma plume is formed in a high temperature and high pressure environment^21,22^. Under these extreme conditions, the nanoparticles can be produced through Coulombic explosion, and surface functionalization of the nanoparticles occurs simultaneously^23^. Hence, femtosecond laser ablation is a convenient method for preparing different nanoparticles, including iron oxide magnetic nanoparticles^24,25^, alloy nanoparticles^26,27^ and C-dots^28–30^. Compared with the bottom-up synthetic strategies, the laser ablation method is more environmentally friendly, benefiting from a decrease in the usage of chemical ligands and the residues of reducing agents. In addition, as the size reduction of the particles into nanostructures can be completed in a short time through laser ablation (tens of minutes typically), the femtosecond laser ablation method for TMDs nanoparticles preparation seems to be timesaving compared with top-down methods such as solvothermal approaches^7,10^.

Herein, we designed a facile route to synthesize the TMDs QDs through femtosecond laser ablation combined with sonication-assisted liquid exfoliation. Using this method, bulk TMDs were first tailored into small nanoparticles using femtosecond laser ablation and then exfoliated into few-layered QDs by ultrasonic processing in liquid. The optical properties and chemical structures were characterized using a transmission electron microscope (TEM), atomic force microscope (AFM), UV-Vis absorption spectroscopy, photoluminescence (PL), X-ray photoelectron spectroscopy (XPS), Fourier transform infrared (FTIR) spectroscopy, and Raman spectroscopy. Meanwhile, the carrier dynamics of the TMDs QDs were investigated using picosecond time-resolved spectroscopy, which showed that the abundance of surface functional groups lead to the TMDs QDs PL. The TMDs QDs fabricated by femtosecond laser ablation exhibited good dispersibility, high purity, bright fluorescence, and low toxicity. In brief, our method is a good candidate for the fabrication of high-quality TMDs QDs as well as boron nitride quantum dots (BNQDs).

## Results and Discussion

**Figure 1:**
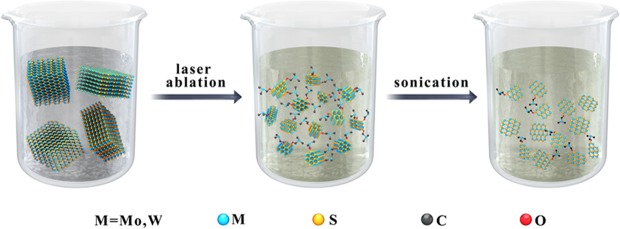
Schematic illustration of the synthetic procedure of TMDs QDs based on femtosecond laser ablation and sonication-assisted liquid exfoliation.

The TMDs QDs were prepared by femtosecond laser ablation combined with sonication-assisted liquid exfoliation of bulk TMDs in NMP, a schematic diagram of the process is shown in Fig. 1, where M represents Mo and W elements. There are two critical steps during the process. First, large bulk MoS_2_ and WS_2_ powders were cut into small multilayer MoS_2_ and WS_2_ nanoparticles by femtosecond laser ablation. Second, the produced multilayer MoS_2_ and WS_2_ nanoparticles were exfoliated into QDs through an ultrasonic exfoliation process. When the two steps were completed, faint yellow solutions containing MoS_2_ and WS_2_ QDs were obtained. Here, NMP was selected as the solvent because its surface energy matched the van der Waals forces of the MoS_2_ and WS_2_ layers^16,31^, which benefits the exfoliation of the MoS_2_ and WS_2_ nanoparticles from the multilayer to the monolayer.

**Figure 2:**
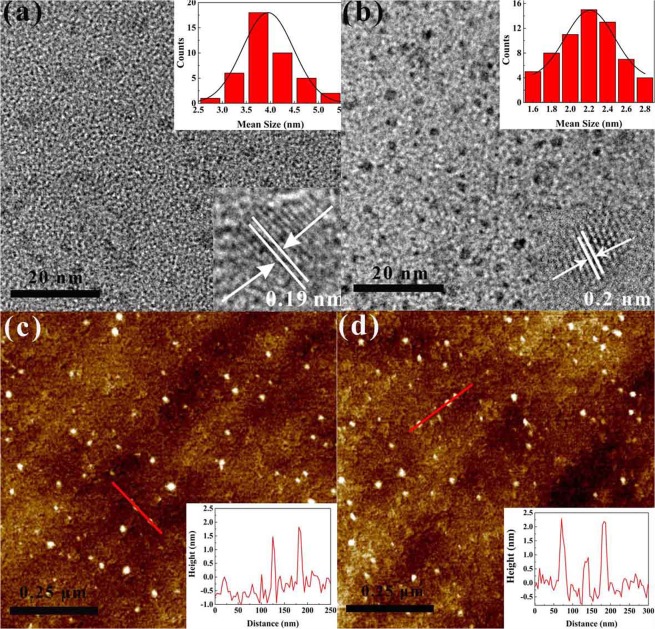
(**a**,**b**) TEM images of the MoS_2_ QDs and WS_2_ QDs. The inset are the size distributions and the HRTEM images of the MoS_2_ QDs and WS_2_ QDs. (**c**,**d**) AFM images of the MoS_2_ QDs and WS_2_ QDs. The inset is the height distribution of MoS_2_ QDs and WS_2_ QDs, marked with a solid red line in (**c**,**d**).

TEM images were used to characterize the microstructure and size distribution of the MoS_2_ and WS_2_ QDs. The TEM samples were prepared by depositing a small droplet of the TMDs QDs solution onto a microscopic copper grid coated with a thin transparent carbon film. As shown in Fig. 2(a,b), the average lateral sizes of the MoS_2_ and WS_2_ QDs were approximately 3.7 nm and 2.1 nm, respectively. The HRTEM images in the inset of Fig. 2(a,b) indicate that both QDs were well-crystallized. The d-spacing of the MoS_2_ QDs was 0.19 nm, corresponding to the (105) facet of the MoS_2_ crystal^1^. A lattice spacing of approximately 0.2 nm could be indexed to the (006) plane of the WS_2_ crystal^32^, indicating that these QDs were MoS_2_ or WS_2_ QDs. To further investigate the morphology and thickness of the as-prepared QDs, AFM measurements of these nanostructures were carried out. The AFM images and the height profile (Fig. 2(c,d)) exhibit typical topographic heights for MoS_2_ and WS_2_ ranging from 1 to 2 nm, corresponding to 1–2 layers of MoS_2_ and WS_2_^14,33^. Due to equipment limitations, the AFM testing was restricted to the current height resolution. These morphological investigations indicated that MoS_2_ and WS_2_ nanoparticles were formed during the laser ablation process and were exfoliated into few-layered QDs after ultrasonic processing in NMP.

**Figure 3:**
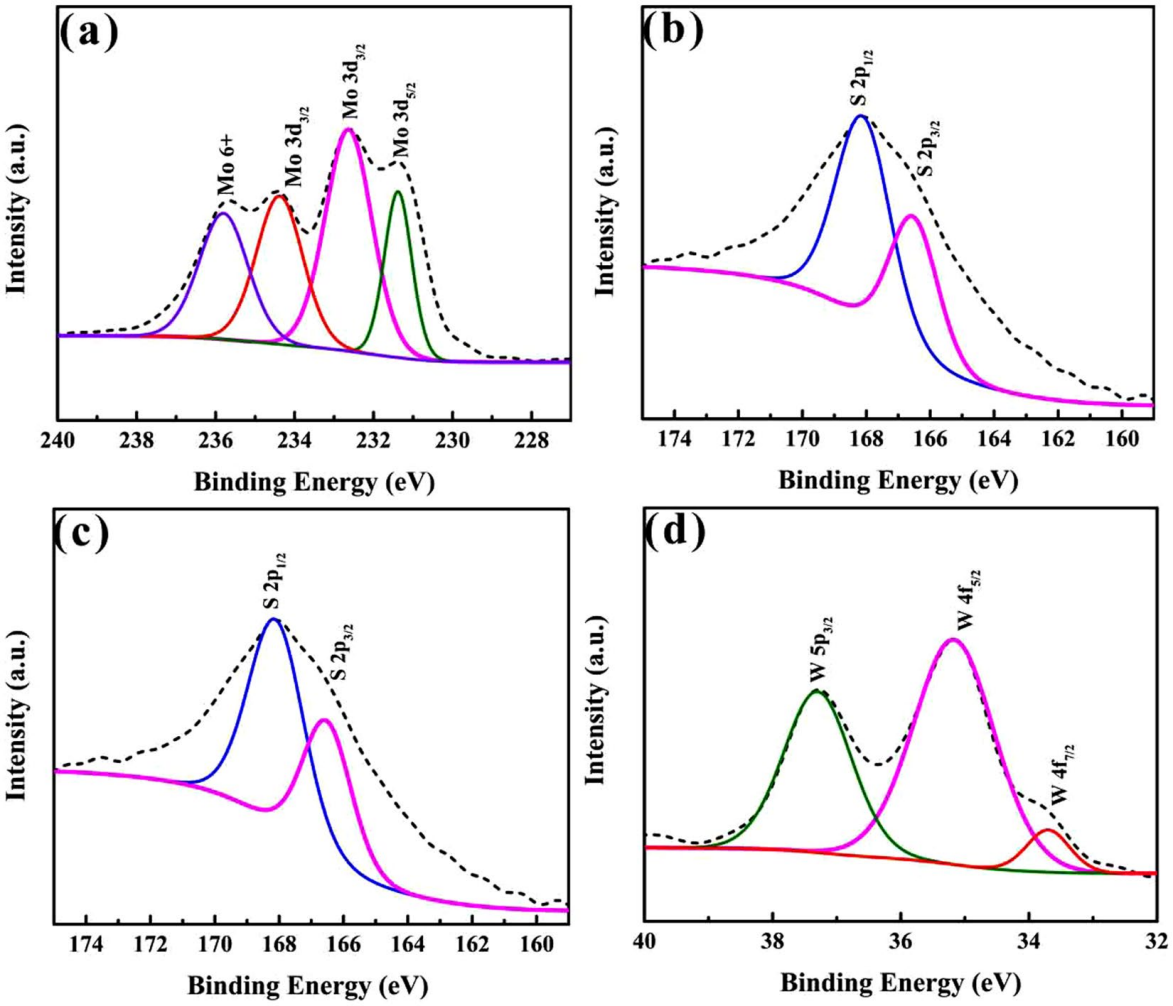
XPS spectra of (**a**) Mo 3d and (**b**) S 2p regions for MoS_2_ QDs and the (**c**) S 2p and (**d**) W 5p and W 4 f regions for WS_2_ QDs.

To explore the chemical structure of the prepared TMDs QDs, XPS measurements of the MoS_2_ QDs and WS_2_ QDs were obtained. The high-resolution spectra of Mo (Fig. 3(a)) showed three peaks at 231, 233 and 234.5 eV, which belonged to Mo^4+^ 3d_5/2_ and Mo^4+^ 3d_3/2_ of 2H-MoS_2_, respectively. Moreover, the existence of Mo^6+^ demonstrated that the Mo edges in the MoS_2_ QDs are oxidized during the preparation process^1,34^. S peaks at 166.5 and 168.3 eV were assigned to S^2−^ 2p_3/2_ and S^2−^ 2p_1/2_ in 2H-MoS_2_ (Fig. 3(b))^3^. As shown in Fig. 3(c,d), the XPS spectra of WS_2_ QDs revealed that the structure of S-W-S was maintained through all of the preparation processes. The S peaks (2p_3/2_ at ~166.5 eV and 2p_1/2_ at ~168.3 eV) in Fig. 3(c) were attributed to the −2 valence state of the S atoms. The peaks for the 4 f level of W atoms that correspond to a bound +4 valence state (WS_2_) are presented in Fig. 3(d). The bands at 33.7, 35.2 and 37.3 eV were assigned to W 4f_7/2_, W 4f_5/2_ and W 5p_3/2_, respectively^35^. The XPS results indicate that functional groups were attached to the surfaces of the MoS_2_ and WS_2_ QDs during the fabrication process. As reported, ionization of the raw materials and solution occurred in the laser ablation process, and a plasma with a high temperature and high pressure was formed^23^. Under these extreme conditions, MoS_2_ and WS_2_ nanoparticles with a size of several nanometres could be produced, and surface functionalization of the nanoparticles occurred simultaneously.

**Figure 4:**
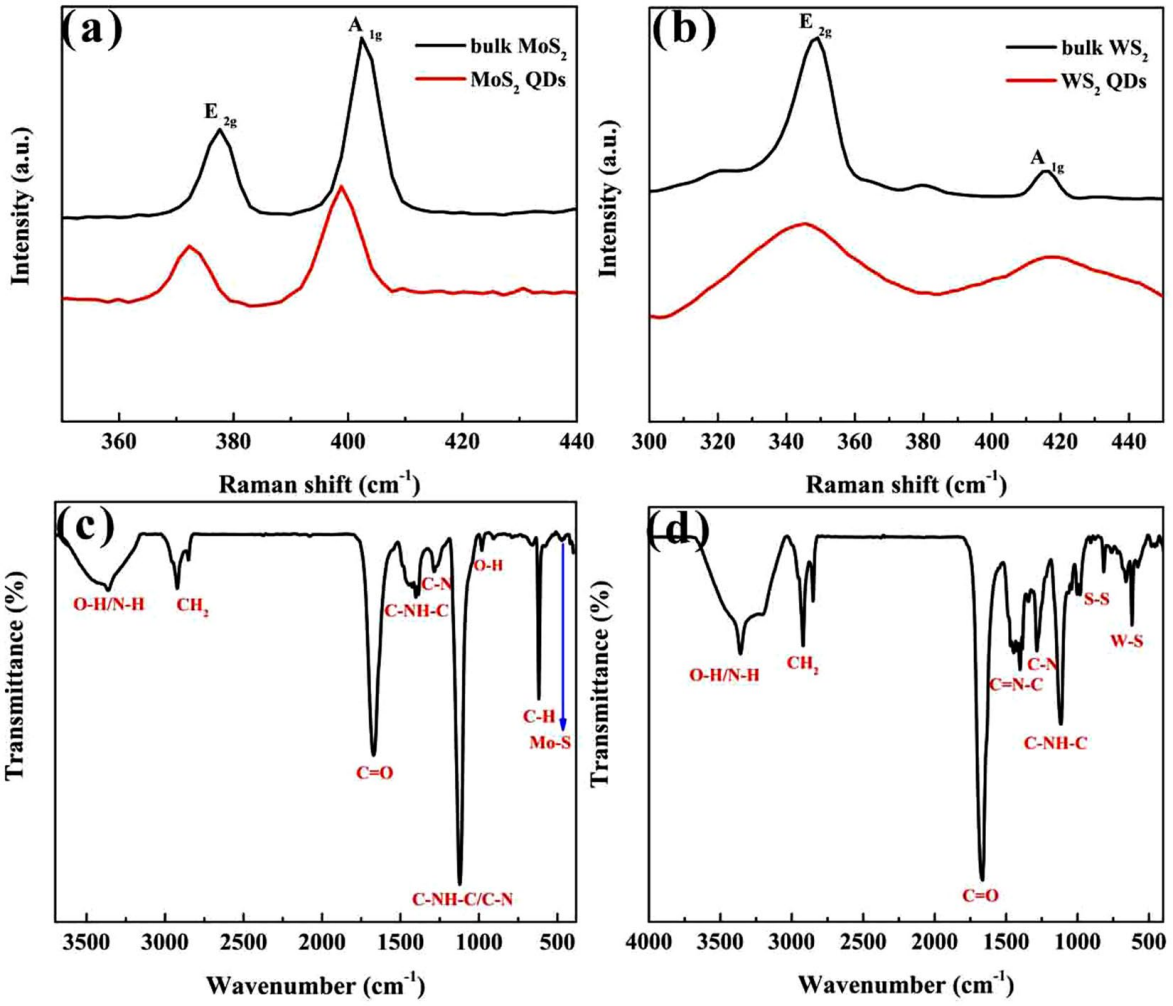
(**a**) Raman spectra of MoS_2_ powder and MoS_2_ QDs. (**b**) Raman spectra of WS_2_ powder and WS_2_ QDs. (**c**) FTIR spectrum of MoS_2_ QDs. (**d**) FTIR spectrum of WS_2_ QDs.

The chemical structures of MoS_2_ and WS_2_ QDs were investigated by Raman and FTIR spectroscopy. In Fig. 4(a), the Raman spectrum of the bulk MoS_2_ powder had two main modes, the A_1g_ (the out-of-plane vibration of the S atoms) and the E_2g_ (the in-plane vibration of the Mo–S bonds) located at 402 and 377 cm^−1^, respectively^5^. The Raman spectrum of the MoS_2_ QDs showed that the A_1g_ peak had blue shifted on the order of 3 cm^−1^, and the peak position of the E_2g_ had also decreased 5 cm^−1^, which was attributed to the A_1g_ softening and E_2g_ stiffening with decreasing layer thickness^3^. In the Raman spectra of WS_2_, the bulk WS_2_ also showed two peaks at approximately 415 (A_1g_) and 348 cm^−1^ (E_2g_) (Fig. 4(b)). For the WS_2_ QDs, the E_2g_ peak blue shifted to 344 cm^−1^, and the A_1g_ peak redshifted to 417 cm^−1^. The blue shift of the E_2g_ was attributed to the reduced long-range Coulomb interactions between the effective charges caused by an increase in the dielectric screening of stacking-induced changes in the interlayer bonding^36^. The shift of the A_1g_ may be caused by a decrease in the interlayer Van der Waals interactions, which results in a weaker restoring force in the vibration as WS_2_ QDs form^32^. The Raman spectra of the MoS_2_ and WS_2_ QDs confirmed that the bulk TMDs were exfoliated into few-layered QDs during the fabrication process.

The FTIR measurements were used to study the surface functional groups of the QDs. The FTIR spectrum of the MoS_2_ QDs (Fig. 4(c)) showed one weak absorption peak at 474 cm^−1^, which could be ascribed to the Mo-S stretching vibration mode of MoS_2_^37^. Figure 4(d) exhibited characteristic absorptions at approximately 821–985 cm^−1^ and 608 cm^−1^, which corresponded to the S-S bond and W-S bond, respectively^36,38^. Apart from the above characteristic peaks, the MoS_2_ QDs and WS_2_ QDs had almost the same FTIR peaks. The appearance of peaks at 3359 cm^−1^ (OH bond stretching), 2924 cm^−1^ (CH_2_ asymmetric stretching), 1673 cm^−1^ (C=O vibration), 1401 cm^−1^ (C-NH-C or C=N-C stretching vibration), 1285 cm^−1^ (C–N stretching frequencies) and 1121 cm^−1^ (C-NH-C or C-N stretching) indicated the attachment of NMP to the QD surface during the femtosecond laser ablation process^5,18,39–41^. In addition, the presence of carboxyl and hydroxyl groups were deemed to be responsible for the good water solubility of the prepared MoS_2_ QDs and WS_2_ QDs.

**Figure 5:**
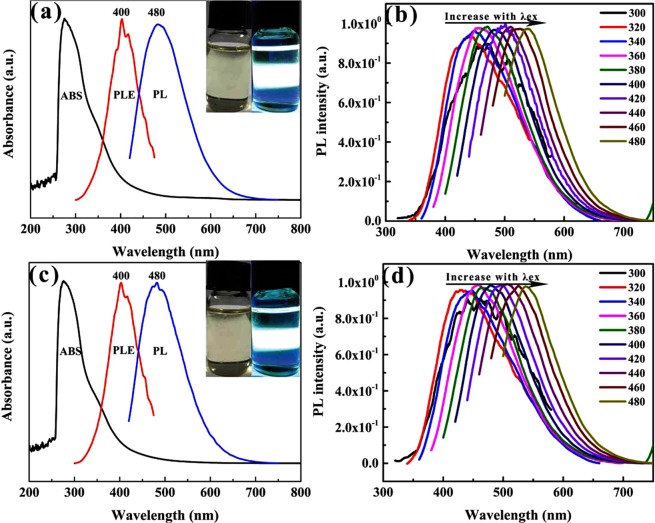
(**a**,**c**) UV–vis ABS (black line), PLE (red line) and PL (blue line) of the MoS_2_ QDs and WS_2_ QDs, respectively. (**b**,**d**) Excitation-dependent PL emission behaviour of the MoS_2_ QDs and WS_2_ QDs, respectively, excited at wavelengths from 300 to 480 nm. The inserts of (**a**,**c**) show photographs of the bulk materials and the corresponding QDs taken under visible (left) and 395 nm UV (right) lights.

UV−vis absorbance, PL excitation (PLE) and PL spectra were obtained to study the optical properties of the MoS_2_ QDs and WS_2_ QDs. The as-prepared MoS_2_ QDs and WS_2_ QDs under visible light were yellowish in colour (as shown by the left inset of Fig. 5(a,c)), while blue-green photoluminescent emission could be observed under UV (395 nm) irradiation (the right inset in Fig. 5(a,c)). As shown in Fig. 5(a), MoS_2_ QDs showed an optical absorption peak at 275 nm with the edge extending to approximately 450 nm, which may be attributed to the functional groups on its surface[3; 4]. Similarly, the WS_2_ QDs had almost the same absorption spectrum. Meanwhile, the strongest emission of the MoS_2_ QDs and WS_2_ QDs occurred at 480 nm under 400 nm light excitation with a Stokes shift of 80 nm. Figure 5(b,d) show that the as-prepared MoS_2_ QDs and WS_2_ QDs all exhibited excitation-dependent PL behaviour, which may be caused by the abundance of surface functional groups of the QDs.

**Figure 6:**
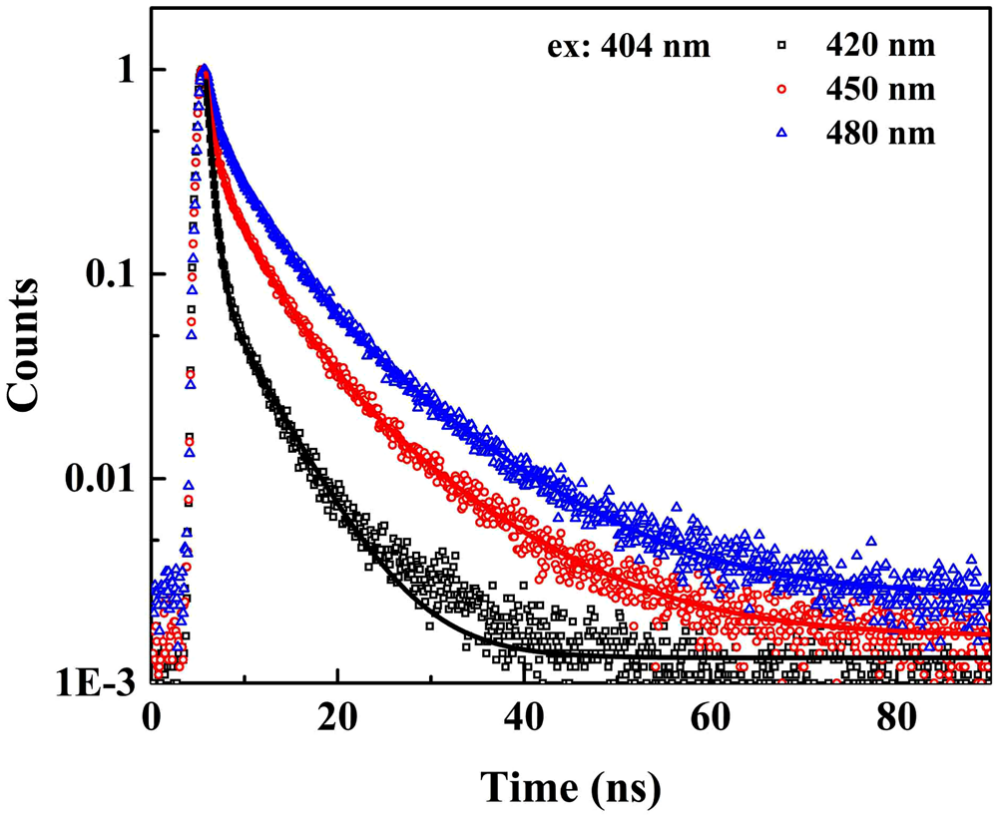
Time-resolved PL spectra of the prepared MoS_2_ QDs at detection wavelengths of 420, 450, and 480 nm under 404 nm excitation.

To study the origin and mechanism of the PL process in MoS_2_ QDs, the NMP solvent was replaced with distilled water for the laser ablation process. As shown in Supplementary Fig. S1, no photoluminescence appeared in the PL spectrum of the prepared MoS_2_ QDs. Because there were no carbon atoms in water, carbon functional groups were not able to form on the MoS_2_ QDs. Therefore, we could infer that the PL of MoS_2_ QDs prepared in NMP originated from its surface functional groups rather than its intrinsic luminescence^42^. Picosecond time-resolved spectroscopy was further used to study the PL mechanism of the prepared MoS_2_ QDs in NMP. The PL emissions were excited using a 404 nm laser, and the temporal behaviour of the emissions at wavelengths of 420, 450, and 480 nm was measured. As shown in Fig. 6, each of the decay curves of these emissions could be well fitted using a double-exponential function, indicating both a fast decay (0.65~0.95 ns) and a slow decay (4.90~7.95 ns). The fitting results are given in Supplementary Table S1. Generally, with increasing emission wavelength, the slow time component in the PL dynamics increased, and the average lifetime of the PL was prolonged. Similar to the PL mechanism in C-dots prepared using laser ablation methods, when the MoS_2_ QDs were excited, there were two pathways for electron-hole recombination in the prepared MoS_2_ QDs: direct radiative recombination of the surface states (a fast decay), and a relaxation of carriers from the intrinsic states of MoS_2_ QDs to the surface states followed by radiative recombination of the surface states (a slow decay)^43^. When the emission wavelength was increased, the lower electron energy levels of the surface states were corresponded, and relaxation from the intrinsic states to the excited surface states was prolonged, causing an increase in the slow time components of the PL lifetime.

Similar to the TMDs QDs, the newly emerged BNQDs have also attracted great attention^44,45^. Unfortunately, the synthesis of BNQDs has also been limited to time-consuming top-down methods due to the difficulty in selecting proper precursors for the bottom-up synthetic strategies^46^. The proposed method in this report, based on femtosecond laser ablation and sonication-assisted liquid exfoliation, was also successfully used to fabricate BNQDs. The experimental details and results are given in the supporting information. TEM images (Supplementary Fig. S2) and XPS (Supplementary Fig. S3) demonstrate the formation of the BNQDs. The PL spectra and the Raman survey (Supplementary Fig. S4) indicate the excellent fluorescence properties of the products.

## Conclusion

In summary, a fast and simple method for the synthesis of high-quality TMDs QDs based on femtosecond laser ablation and sonication-assisted liquid exfoliation is proposed. The bulk MoS_2_ and WS_2_ were cut into small nanoparticles by femtosecond laser ablation, and the ultrasonic process exfoliated these nanoparticles into MoS_2_ QDs and WS_2_ QDs. By analysing the results of TEM, AFM, XPS, FTIR and PL, we found that the prepared MoS_2_ QDs and WS_2_ QDs were few layered and exhibited good optical properties. To study the origin and mechanism of the PL process in MoS_2_ QDs, time-resolved PL was also investigated. In addition, our work also provides a fast, low-cost, and simple synthetic strategy for the synthesis of transitional metal dichalcogenides QDs and other 2D nanomaterials.

## Methods

### Materials

Bulk hexagonal boron nitride (hBN), bulk MoS_2_ powders, bulk WS_2_ powders and N-methyl-2-pyrrolidone (NMP, 99.5%) were commercially purchased from Aladdin Industrial Co. Ltd. (Shanghai, China). All materials were of analytical grade and used without further purification.

### Preparation of TMDs QDs

Using the MoS_2_ QDs as an example, the MoS_2_ QDs were prepared using the following procedures. A total of 3.2 mg of MoS_2_ powder was dispersed into 40 mL of NMP solution, and the mixture was sonicated for 2–3 minutes to obtain a uniform distribution. Next, 10 mL of the above solution was placed into a glass beaker (outside diameter × height: 25 mm × 35 mm) for ablation. By focusing with a lens (focal length 100 mm), a femtosecond laser with a wavelength of 800 nm that was produced by a Ti:sapphire laser (with 80 fs pulse duration, 400 mW laser power, and 1 kHz repetition rate) was directed into the solution for approximately 0.5 h. During laser irradiation, a magnetic stirrer was used to prevent gravitational settling of the initial powder. After laser ablation, the solution was centrifuged for 20 min at 12000 rpm to remove large MoS_2_ particles. The supernatant was collected and processed by ultrasound for 2 h with 500 mW power. During the sonication process, an ice/water system was used to maintain a temperature of 10 °C. After sonication, the prepared MoS_2_ QDs contained in the supernatant were collected for use. The fabrication process of the WS_2_ QDs was similar to that of the MoS_2_ QDs. The yield of QDs from the powder suspensions was seriously affected by the ablation conditions, such as the pulse energy, irradiation time and spot size^23,47^. In our experiments, the yield of the QDs for 30-min laser ablation was estimated to be approximately 11%, which is comparable with that reported in previous studies^7^.

### Instrumentation

The PL spectrum measurements were conducted with a spectrometer (Omni-λ, China). The UV-vis absorption spectra were obtained from a spectrophotometer (UV-2600, China). TEM and high resolution TEM (HRTEM) images of the BNQDs were obtained using a high-resolution transmission electron microscope (JEM-ARM200F, Japan). XPS experiments were carried out with an X-ray photoelectron spectrometer (ESCALAB Xi+, USA). The AFM images were obtained using an atomic force microscope (DIMENSION IOON, Germany). The Raman spectra were acquired from a Raman System (HR800, France) with a 532 nm laser excitation. FTIR spectroscopy was performed with a time-resolution infrared spectrometer (Vetex70, Germany) using the KBr pellet method. The time-resolved PL spectra of the BNQDs were monitored with a time-correlated single-photon counting system (FLSPP20, UK) (excited by picosecond pulsed LDs, a time resolution of 100 ps, pulse duration: <850 ps, repetition rate: 10 MHz).

### “Ethics”

We were not required to complete an ethical assessment prior to conducting our research, and no permissions were required prior to conducting our research.

## Supplementary information

Dataset 1

## Data Availability

All of the data generated or analysed during this study are included in this published article and its Supplementary Information files.
