# Supplementary material for: Fabrication of transition metal dichalcogenides quantum dots based on femtosecond laser ablation
Source: Sci Rep. 2019 Feb 27;9:2931. doi: 10.1038/s41598-019-38929-5 (PMC6393534; doi:10.1038/s41598-019-38929-5)
Supplement: Supplementary file 1 — Dataset 1 [file 41598_2019_38929_MOESM1_ESM.pdf]

Supplementary Information

Fabrication of transition metal dichalcogenides quantum dots based on femtosecond laser ablation

Yanmin Xu, Lihe Yan*, Xiaoyu Li, Huanhuan Xu

Key Laboratory for Physical Electronics and Devices of the Ministry of Education and Shaanxi Key Lab of Information Photonic Technique, School of Electronics and Information Engineering, Xi'an Jiaotong University, Xi'an 710049, China,

Yanmin Xu email: 2903542602@qq.com

Lihe Yan email: liheyan@mail.xjtu.edu.cn

Xiaoyu Li email: 1597433391@qq.com

Huanhuan Xu email: 316788792@qq.com

Corresponding author email: liheyan@mail.xjtu.edu.cn

Corresponding author telephone number: +86-029-82665670

Preparation and characterizations of BNQDs

BNQDs was also prepared using femtosecond laser ablation and sonication-assisted exfoliation technology. A certain quality of bulk hBN powders in NMP solvent were suffered from femtosecond laser ablation. After laser ablation, multilayer nanoparticles were gained. Those nanoparticles were centrifuged to remove large particles and the supernatant was collected and processed by ultrasonic exfoliation process. After sonication, the prepared BNQDs contained in the supernatant were collected for use. For some reasons, the chemical and optical properties of QDs were preliminary characterized using TEM, AFM, Raman, XPS and PL spectra.

As shown in Supplementary Fig. S2 (a), the TEM images of BNQDs showed they were all monodispersed. The HRTEM image of the BNQDs was well-crystallized with a lattice spacing of 0.22 nm, corresponding to the (100) face of the hBN crystal[1; 2]. The AFM image and the height profile (Supplementary Fig. S2(c) and (d)) exhibited a typical topographic height of the BNQDs ranging from 1 to 1.5 nm, corresponding to

1–2 BN layers[1].

Raman spectrum of the as-prepared BNQDs showed an E_{2g} phonon mode at around 1367 cm^{-1}, being similar with that of the hBN raw materials[3; 4]. The measured full width half maximum (FWHM) of BNQDs (12.48 cm^{-1}) was larger than that of bulk hBN (10 cm^{-1}), which could be attributed to the laser ablation[5]. As shown in Supplementary Fig. S3 (b), four same peaks at 189.67, 284.45, 397.43, 531.46 eV were revealed by the XPS survey spectrum of hBN and BNQDs, evidencing the presence of B, C, N, and O elements, respectively. Supplementary Fig. S3 (c) and Supplementary Fig. S3 (d) showed the high-resolution XPS spectra of B 1s and N 1s, respectively. In Supplementary Fig. S3 (c), the B 1s spectra could be fitted using three peaks at 186.8, 188.3, and 190.2 eV, which corresponded to B-C, B-N, and B-O bonding, respectively[6; 7]. Moreover, the high-resolution N 1s XPS spectrum was well fitted using one peak at approximately 397.8 eV, indicating the existence of N-B bonds[7]. The XPS results confirmed the synthesis of the BNQDs based on femtosecond laser ablation. The presence of B-C and B-O bonds indicated that functional groups were linked on the surface of BNQDs in the fabrication process.

Similar with the prepared TMDs QDs, the PL spectra (Supplementary Fig. S4 (b)) of BNQDs was exhibited the excitation-dependent behavior, which may related to the surface functional groups of QDs. In Supplementary Fig. S4 (c), the FTIR spectrum of BNQDs confirmed the existence of abundant surface functional groups on the prepared BNQDs.

Figures

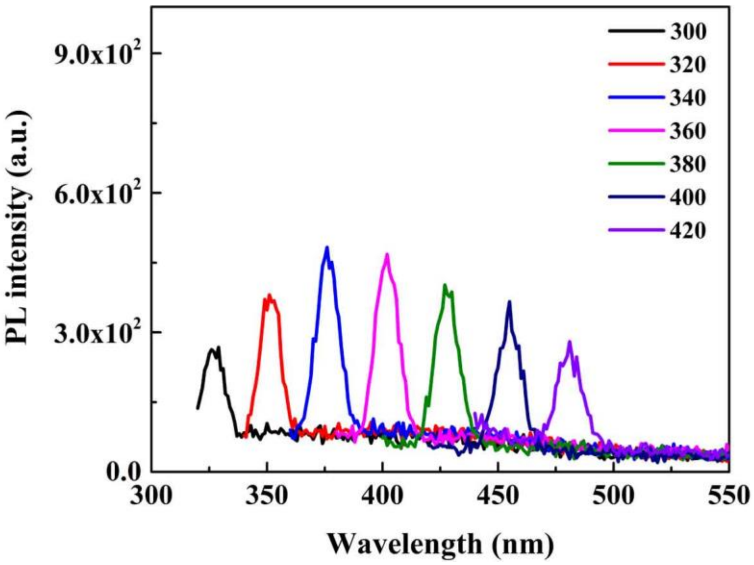

Figure S1 Fluorescence emission spectrum of the MoS_2 QDs which prepared in distilled water recorded at excitation wavelength from 300 nm to 420 nm.

Table S1 Double-exponential fitting for time-resolved PL spectrum of the MoS_2 QDs at various detection wavelengths when excited at 404 nm.

λ_{ex}	λ_{em}	τ_1 (ns)	τ_2 (ns)	τ_{ave} (ns)
404 nm	420 nm	0.49 (89%)	3.46 (11%)	0.82
	450 nm	0.58 (68%)	5.56 (32%)	2.17
	480 nm	0.54 (54%)	5.78 (46%)	2.95

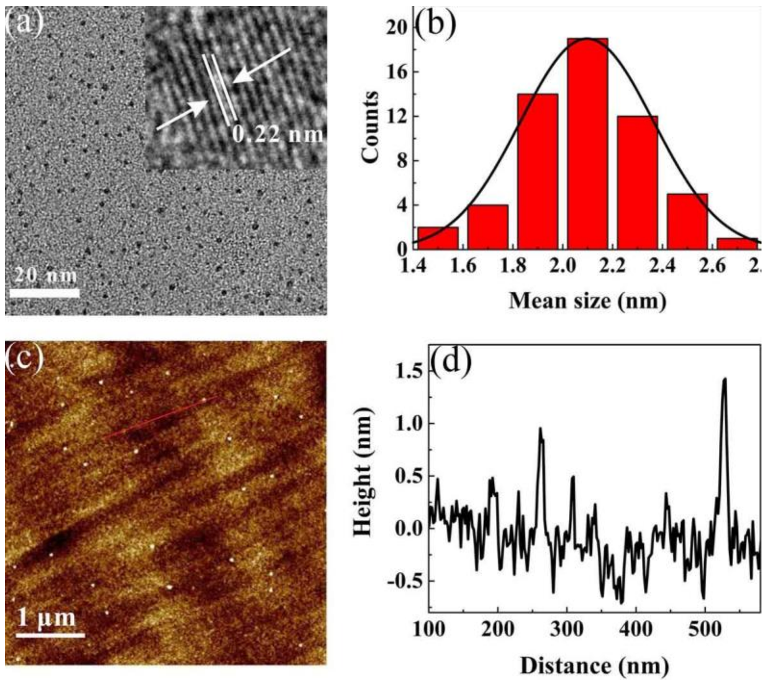

Figure S2 (a) TEM image of the BNQDs; the inset is the HRTEM image of BNQDs. (b) Size distribution of BNQDs. (c) AFM image of BNQDs. (d) Corresponding height distribution of BNQDs, marked with a solid red line in (c).

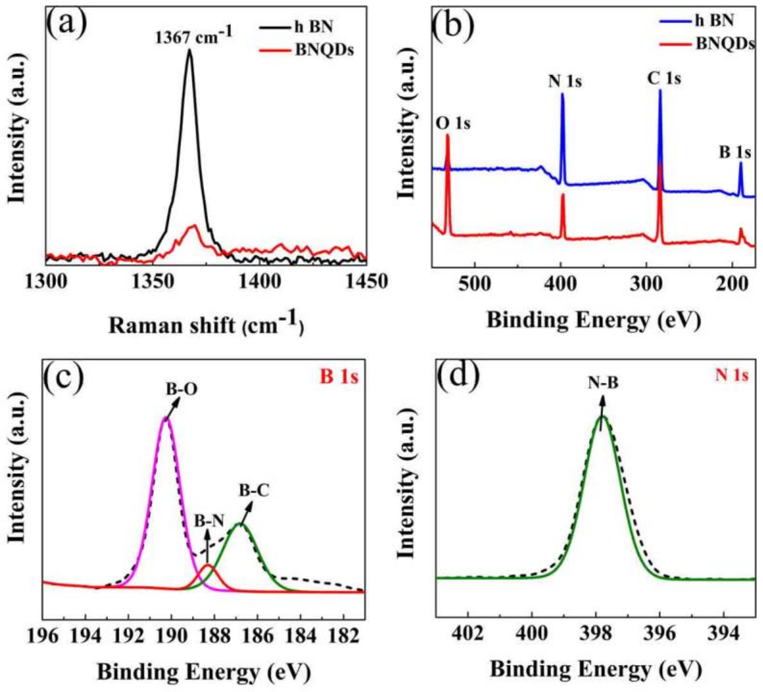

Figure S3 (a) Raman spectra of hBN powder and BNQDs. (b) XPS spectra of hBN powder and BNQDs. (c) high-resolution B 1s and (d) N 1s spectra of BNQDs.

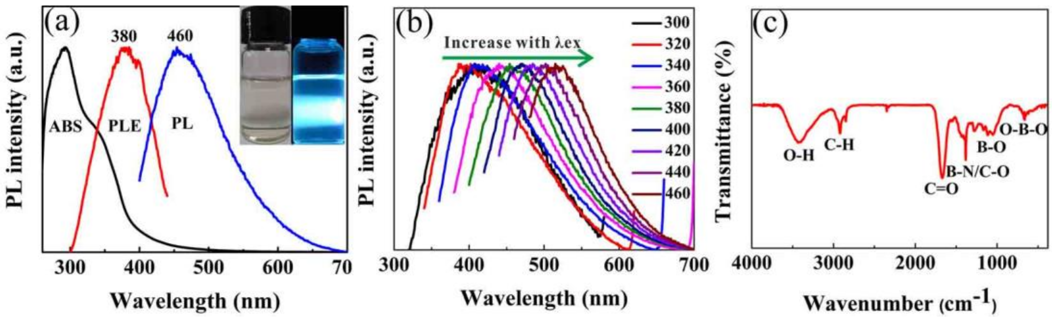

Figure S4 Optical properties of the prepared BNQDs in NMP. (a) UV–vis absorbance ABS (balck line), PLE (red line); (b) excitation-dependent PL emission behavior of BNQDs, excited at wavelengths from 300 to 460 nm; (c) FTIR spectrum. The insert of (a) shows corresponding photographs of hBN and the BNQDs taken under visible (left) and 395 nm UV (right) lights.

References:

1 Tay, R. Y. et al. Growth of large single-crystalline two-dimensional boron nitride hexagons on electropolished copper. *Nano. Lett*. **14,** 839-846 (2014).

2 Gorbachev, R. V. et al. Hunting for monolayer boron nitride: optical and raman signatures. *Small* **7,** 465-468 (2011).

3 Lin, L. et al. Fabrication and Luminescence of Monolayered Boron Nitride Quantum Dots. *SMALL*. 10, 60-65 (2014).

4 Arenal, R. et al. Raman spectroscopy of single-wall boron nitride nanotubes. *Nano. Lett*. **6,** 1812-1816 (2006).

5 Thangasamy, P., Santhanam, M. & Sathish, M. Supercritical fluid facilitated disintegration of hexagonal boron nitride nanosheets to quantum dots and its application in cells imaging. *ACS Appl. Mater. Inter*. **8,** 18647-18651 (2016).

6 Huo, B. B. et al. One-step synthesis of fluorescent boron nitride quantum dots via a hydrothermal strategy using melamine as nitrogen source for the detection of Ferric ions. *Langmuir* **33,** 10673-10678 (2017).

7 Fan, L. D. et al. Facile microwave approach to controllable boron nitride quantum dots. *J. MATER. Sci*. **52,** 13522-13532 (2017).
